# A study of the chest imaging findings of adult patients with COVID-19 on admission to a tertiary hospital in Johannesburg, South Africa

**DOI:** 10.4102/sajid.v37i1.449

**Published:** 2022-08-30

**Authors:** Ashleigh A. Ord, Jarrod Zamparini, Liam Lorentz, Ashesh Ranchod, Halvani Moodley

**Affiliations:** 1 Department of Diagnostic Radiology, Faculty of Health Sciences, University of the Witwatersrand, Johannesburg, South Africa; 2 Department of Internal Medicine, Faculty of Health Sciences, University of the Witwatersrand, Johannesburg, South Africa; 3 Department of Internal Medicine, Charlotte Maxeke Johannesburg Academic Hospital, Johannesburg, South Africa; 4 Department of Radiology, Capital Radiology, Pretoria, South Africa; 5 Department of Radiology, NRS Incorporated Netcare N17 Private Hospital, Springs, South Africa; 6 Department of Diagnostic Radiology, Charlotte Maxeke Johannesburg Academic Hospital, Johannesburg, South Africa

**Keywords:** COVID-19, chest imaging, chest radiograph, chest CT, HIV

## Abstract

**Background:**

South Africa has experienced multiple waves of the coronavirus disease 2019 (COVID-19) with little research documenting chest imaging features in an human immunodeficiency virus (HIV) and tuberculosis (TB) endemic region.

**Objectives:**

Describe the chest imaging features, demographics and clinical characteristics of COVID-19 in an urban population.

**Method:**

Retrospective, cross-sectional, review of chest radiographs and computed tomographies (CTs) of adults admitted to a tertiary hospital with severe acute respiratory syndrome coronavirus 2 (SARS-CoV-2) infection, between 01 May 2020 and 30 June 2020. Imaging was reviewed by three radiologists. Clinical parameters and laboratory data were analysed.

**Results:**

A total of 113 adult patients with a mean age of 46 years and 10 months were included. A total of 113 chest radiographs and six CTs were read. Nineteen patients were HIV-positive (16.8%), 40 were hypertensive and diabetic (35.4%), respectively, and one had TB (0.9%). Common symptoms included cough (*n* = 69; 61.6%), dyspnoea (*n* = 60; 53.1%) and fever (*n* = 46; 40.7%). Lower zone predominant ground glass opacities (58.4%) and consolidation (29.2%) were most frequent on chest radiographs. The right lower lobe was most involved (46.9% ground glass opacities and 17.7% consolidation), with relative sparing of the left upper lobe. Bilateral ground glass opacities (66.7%) were most common on CT. Among the HIV-positive, ground glass opacities and consolidation were less common than in HIV-negative or unknown patients (*p* = 0.037 and *p* = 0.05, respectively).

**Conclusion:**

COVID-19 in South Africa has similar chest imaging findings to those documented globally, with some differences between HIV-positive and HIV-negative or unknown patients. The authors corroborate relative sparing of the left upper lobe; however, further research is required to validate this currently unique local finding.

## Introduction

Since the first documented case of severe acute respiratory syndrome coronavirus 2 (SARS-CoV-2) in the country in March 2020, South Africa has experienced multiple waves of coronavirus disease 2019 (COVID-19) despite the initiation of vaccination programmes countrywide in February 2021, beginning with healthcare workers and progressing to the general public in an age-staged rollout. As of the end of June 2022, there have been over 3.9 million and 101 000 documented infections and deaths, respectively.^1^ Globally, over 554 million individuals have been infected, with an associated mortality rate of approximately 1.1%.^2^

The described symptoms of COVID-19 are well documented, with pneumonia being one of the most serious manifestations, more commonly encountered in the elderly and those with comorbid diseases.^3,4,5^ Pneumonia in these patients can progress to respiratory failure requiring mechanical ventilation^4^ – a scenario that is not only fatal in a proportion of cases but can also quickly overwhelm the intensive care units (ICUs) and high-care facilities of hospitals. A number of repurposed drugs such as dexamethasone and tocilizumab have shown a mortality benefit in the treatment of severe COVID-19,^6^ and several vaccines have been rolled out globally which seek to limit the spread of the virus, prevent disease and/or limit disease progression to severe illness.^7^

The diagnosis of COVID-19 is suspected based on a clinical history of common symptoms, a positive contact or a suspected positive contact and typical clinical examination findings.^4,5^ These patients commonly have a raised C-reactive protein (CRP), lactate dehydrogenase and lymphopaenia.^4,5,8^ The gold standard for definitive diagnosis is laboratory testing of respiratory tract specimens by means of reverse transcription polymerase chain reaction (RT-PCR) assays.^4,8^

At the outset of the pandemic, chest imaging features, in addition to symptoms and exposure history, were used to make the clinical diagnosis of COVID-19 in China.^8^ The current role of chest imaging (ultrasound, radiographs and computed tomography [CT]) is for the monitoring of patients for disease progression and the identification of complications thereof. Chest CT is the gold standard for imaging in patients with COVID-19,^9^ with a sensitivity of 67% – 100%,^10,11^ and in some studies with smaller sample sizes up to 98% sensitivity.^12^ The specificity of findings on CT in COVID-19 is relatively low at 25%.^11^ Comparatively, the sensitivity of chest radiographs in the detection of COVID-19 has been shown to increase with time after symptom onset, ranging from 55% to 79%,^13^ with serial radiographs improving the diagnostic accuracy to one that approaches that of a CT chest.^13^ The specificity of chest radiographs ranges from 73% to 80% and has been found to decrease over time as the disease progresses.^13^

Given the resource limitations of the public health sector in South Africa, the routine use of CT to evaluate the thoracic manifestations of COVID-19 is unrealistic from a financial perspective, impractical given the limited number of CT scanners and unfeasible given the infection control issues related to the transporting of infected patients to and from the CT suites,^14,15^ as well as the resources required for disinfection thereafter. Mobile chest radiography is consequently used as first-line imaging in this setting for patients with confirmed and suspected COVID-19.

The literature documenting the thoracic imaging findings of COVID-19 has rapidly advanced throughout the various global peaks. Ground glass opacification and consolidation have been found to be the major findings on both CT and chest radiographs with a predominantly peripheral distribution^8,11,16^ and bilateral lung involvement.^11,16^ Other less common findings include crazy paving, linear opacities, septal thickening, pleural effusions, nodules, reverse atoll sign, traction bronchiectasis and architectural distortion, although there is marked heterogeneity across studies with regard to the reported percentages of these findings.^11^ Studies performed during the first wave of COVID-19 in Europe also noted a high percentage of lymphadenopathy (59%) – an uncommon finding in other populations.^8^ Many studies document an absence of pleural effusions,^17,18,19^ while a study performed in South Africa found pleural effusions to be the third most common finding in their cohort,^20^ with limited information provided on the presence of background chronic lung disease across all studies. In another recent South African study, Buckley et al. described the relative sparing of the left upper lobe on the chest radiographs of patients admitted to ICU, a finding that has, to the authors’ knowledge, not been previously documented.^21^

This study describes the chest imaging findings (chest radiographs and CTs), demographics and clinical characteristics of COVID-19 in an urban population on admission to a tertiary healthcare facility during the first wave of the pandemic.

## Research methods and design

**FIGURE 1:**
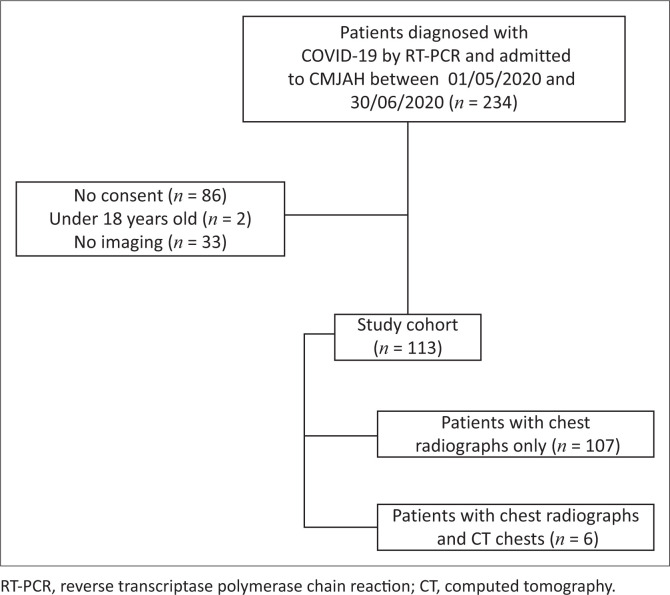
Cohort selection flowchart.

A retrospective, cross-sectional study was performed at the Charlotte Maxeke Johannesburg Academic Hospital (CMJAH), a tertiary hospital in the Gauteng province. All patients 18 years and older, with RT-PCR-confirmed SARS-CoV-2 and either a chest radiograph, CT chest or both, admitted to CMJAH between 01 May 2020 and 30 June 2020 were included in the study. Patients under the age of 18 years, those with pending SARS-CoV-2 results at the end of the data collection period and cases with unevaluable imaging due to poor technical factors were excluded from the study (Figure 1). Each included patient had at least one chest radiograph during admission, and only the first radiograph was used, resulting in 113 chest radiographs for review. Only six patients had CT chests done during the study period.

The clinical data of all patients admitted to the hospital with COVID-19 were recorded on a dedicated database. The relevant demographic and clinical features of the study participants were collected, exported and anonymised by the principal investigator.

The corresponding chest imaging was obtained from the hospital picture archiving and communication system (PACS) – Philips Intellispace PACS Radiology version 4.4.532.1 (Koninklijke Philips Electronics N.V, the Netherlands). All digital chest radiographs were acquired in the standard erect posteroanterior position, anteroposterior position or in the supine position if mobile radiography was used. Chest CTs were performed on one of three multidetector scanners (Siemens SOMATOM Definition AS 64 slice CT scanner, Phillips Ingenuity 64 slice CT scanner or Phillips Ingenuity 128 slice CT scanner) using standard thoracic imaging protocols with intravenous iohexol (Omnipaque, GE Healthcare). These images were then evaluated independently by three radiologists, and a majority decision was used as the final interpretation. All readers were blinded to the participant identification as well as clinical background. The imaging studies were assessed according to a checklist that made use of the reporting terminology as defined in the Fleischner Society: Glossary of terms for thoracic imaging.^22^ A central distribution was defined as the inner two-thirds of the lung and a peripheral distribution as the outer one-third of the lung. On chest radiographs, the upper zone was defined as extending from the superior hilar markings to the apices, the middle zone extending from the superior hilar markings to the inferior hilar markings and the lower zone from the inferior hilar markings to the costophrenic sulcus. Detailed checklists for the readers for chest radiographs and chest CT are included as Appendix 1 and Appendix 2, respectively.

The readers’ findings and the corresponding clinical information were analysed using SAS Software Version 9.2 (SAS Institute Inc., United States of America). Descriptive statistics were calculated and numerical or categorical data reported using means with standard deviations or medians with interquartile ranges if the *p*-value was < 0.05 using the Shapiro–Wilk test to investigate the data distribution. Discrete or categorical data were reported using frequencies and percentages.

### Ethical considerations

Ethics approval for this study was granted by the University of the Witwatersrand, Human Research Ethics Committee (Medical) (ref. no. M200658 R14/49).

## Results

### Clinical characteristics

Five hundred and eight chest radiographs and 15 CT chests were performed at CMJAH during the study period. A total of 113 patients were included in the study, of which 58 (51.3%) were male. The patients’ ages ranged from 19 to 87 years with a mean (standard deviation [s.d.]) of 46 years and 10 months (14.3). Six patients were pregnant (9% of the female patients). One hundred and two patients (90%) were symptomatic for COVID-19, while the remaining 11 (9.7%) were admitted as they were unable to safely self-isolate or for an unrelated indication, and were incidentally found to have SARS-CoV-2 infection. The most common presenting symptoms were cough (61%) and dyspnoea (53%), followed by fever (40.7%) (Table 1a). C-reactive protein was recorded for 109 of the patients and was elevated above the normal range in 96 patients (88%) with a median of 101.0 mg/L (interquartile range [IQR]: 39.0–199.0). The lymphocyte count was recorded for 86 patients with a median count of 1.2 × 10^9^/L (IQR: 0.8–1.8). The median haemoglobin for the cohort was 13.7 g/dL (IQR: 12.1–15.0).

**TABLE 1a:** Symptoms on admission (*n* = 113).

Symptoms on admission	*n*	%
Cough	69	61.6
Dyspnoea	60	53.1
Fever	46	40.7
Sore throat	22	19.5
Myalgia	21	18.6
Malaise	18	15.9
Fatigue	17	15.0
Asymptomatic	11	9.7
Headache	9	8.0
Diarrhoea	6	5.3

The most common comorbidities were hypertension (*n* = 40 patients, 35.4%) and type 2 diabetes mellitus (*n* = 34 patients, 30.1%). Nineteen patients (16.8%) were known to be human immunodeficiency virus (HIV)-positive with CD4 counts ranging from 2 cells/µL to 1240 cells/µL and viral loads ranging from < 20 copies/mL to 56 900 copies/mL. The HIV status of 69 patients was unknown. Two patients had post-tuberculosis bronchiectasis and one patient was being treated for active pulmonary tuberculosis (TB). Two patients had comorbid malignant disease, namely squamous cell carcinoma of the mouth and acute myeloid leukaemia. Further demographic data related to comorbidities are summarised in Table 1b.

**TABLE 1b:** Comorbid conditions (*n* = 113).

Comorbid conditions	*n*	%
Hypertension	40	35.4
Diabetes mellitus	40	35.4
Type 1 DM	1	0.9
Type 2 DM	34	30.1
HIV	19	16.8
Chronic kidney disease	7	6.2
On dialysis	5	71.4†
COPD	4	3.5
Ischaemic heart disease	3	2.7
Cancer	2	1.8
Chronic lung disease	2	1.8
Post-TB bronchiectasis	2	1.8
Asthma	1	0.9
Previous stroke	1	0.9
Active TB	1	0.9
Portal hypertension	1	0.9
Autoimmune hepatitis	1	0.9
Ankylosing spondylitis	1	0.9
Cor pulmonale	1	0.9
Dementia	1	0.9
Graves’ disease	1	0.9
Peripheral vascular disease	1	0.9
Schizophrenia	1	0.9
SLE	1	0.9

DM, diabetes mellitus; HIV, human immunodeficiency virus; CKD, chronic kidney disease; COPD, chronic obstructive pulmonary disease; TB, tuberculosis; SLE, systemic lupus erythematosus.

† , Percentage of patients with CKD.

### Chest radiograph findings

**FIGURE 2:**
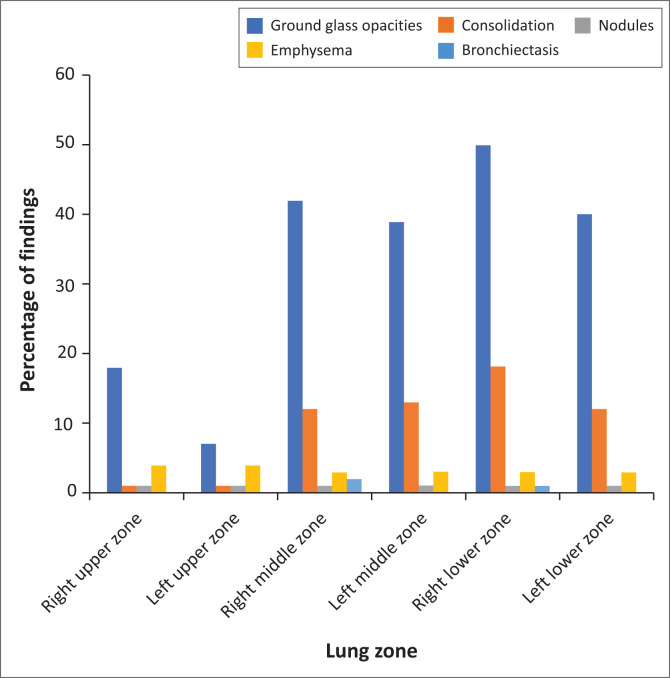
Proportions of the zonal distribution and description of pathological findings on chest radiographs. The right lower zone was found to have the highest frequency of described COVID-19 changes, while the left upper zone was relatively spared.

**FIGURE 3:**
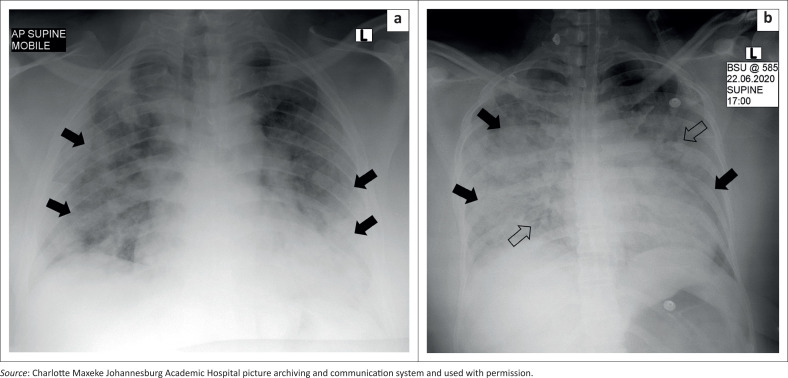
(a) Chest radiograph of a 39-year-old man with type 2 diabetes mellitus and hypertension who presented with a cough, dyspnoea and fever. There are typical findings of predominantly peripheral, patchy ground glass opacities bilaterally (black arrows). (b) Chest radiograph of a 48-year-old woman with asthma, hypertension and severe COVID-19 pneumonia. Bilateral, multi-zonal, patchy, ground glass opacities (black arrows) and consolidation with air bronchograms (open arrows).

**FIGURE 4:**
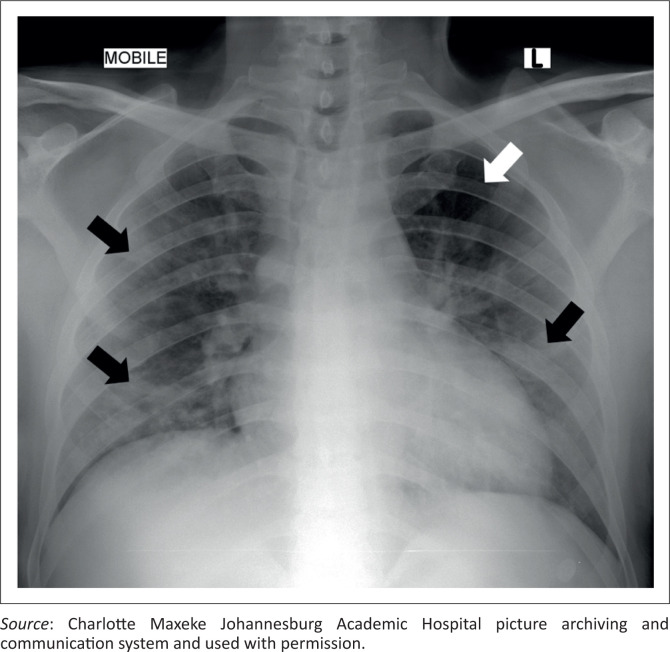
Chest radiograph of a 52-year-old man with hypertension, diabetes mellitus and chronic kidney disease who presented with fatigue and malaise. There are patchy, peripheral and central ground glass opacities bilaterally (black arrows), with sparing of the left upper zone (white arrow).

Seventy-six (67%) of the 113 radiographs were abnormal. The distribution and description of pathological findings are detailed in Figure 2. The predominant findings were ground glass opacities (58.4%), followed by consolidation (29.2%). Ground glass opacities were bilateral in 50 (56.5%) radiographs, with bilateral consolidation present in 11 (12.4%) radiographs (Figure 3). The distribution of ground glass opacities was lower zone predominant, with a decreasing frequency in the mid and upper zones. The distribution of consolidation was similar. The left upper zone was the least affected zone (Figure 4), with eight (7.1%) radiographs showing ground glass opacities and one (0.9%) study with consolidation. The right lower zone was the most affected lung zone, with 46.9% ground glass opacities and 17.7% consolidation. Further findings such as diffuse nodules were detected in one (0.9%) study, bronchiectatic changes in two (1.8%) studies and emphysema in five (4.4%) studies with pleural effusions in two (1.8%) studies. Cavitation, pneumothoraces and lymphadenopathy were not detected.

Half of the small pregnant cohort of patients had normal radiographs. Two patients (30.0%) had bilateral lower zone predominant ground glass opacities, one patient (16.7%) had unilateral right-sided lower zone opacities and one patient (16.7%) had both bilateral lower zone ground glass opacification as well as consolidation in a similar distribution.

### Chest computed tomography findings

**FIGURE 5:**
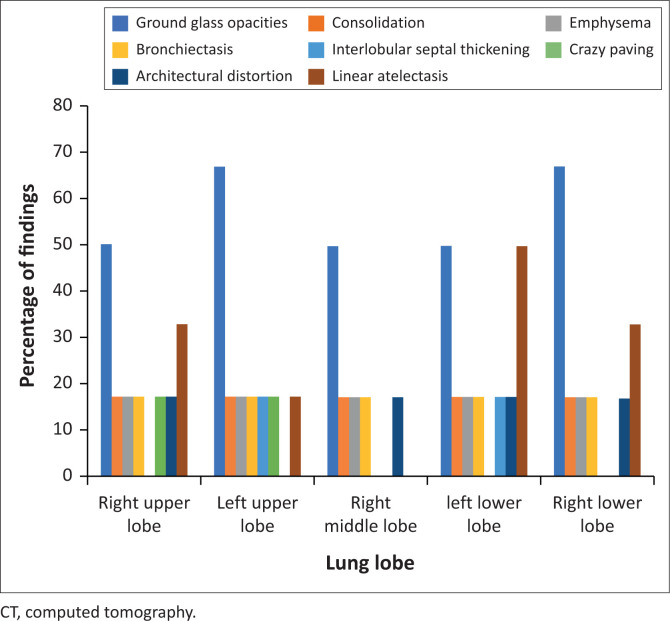
Proportions of the lobar distribution and description of pathological findings on the chest CTs. Ground glass opacities and consolidation were the most common findings.

**FIGURE 6:**
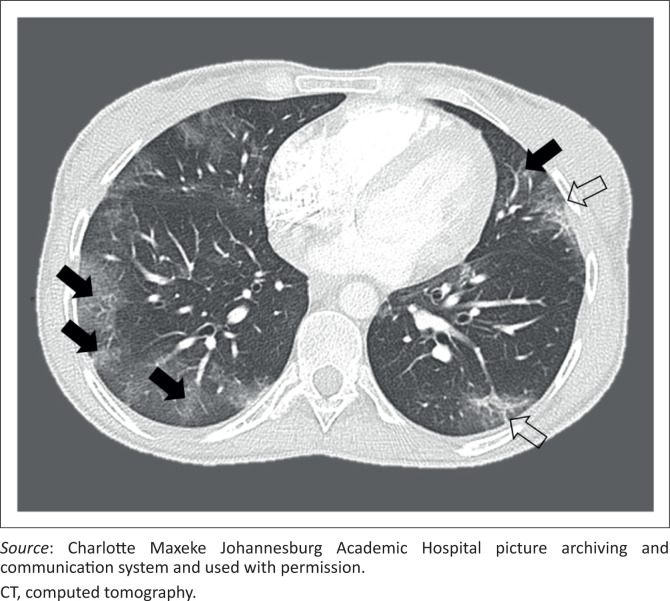
Axial CT lung window of a 31-year-old female patient with no comorbidities, who presented with dyspnoea, fever and a sore throat. Typical findings of peripheral patchy ground glass opacities in the lower lobes (black arrows) with interlobular septal thickening (open arrows).

Four of the six CT chests (66.7%) were abnormal, the distribution and description of which are detailed in Figure 5. The most common finding was bilateral ground glass opacities in four (66.7%) of the studies (Figure 6). One study (16.7% of included CTs) demonstrated consolidation in all lobes in a predominantly peripheral and anterior distribution. Other COVID-19-related findings were interlobular septal thickening, crazy paving and architectural distortion, all present in one study each. The halo sign and reverse halo sign were not detected. Linear atelectasis was documented in two studies, as well as bronchiectatic change and emphysema present in one study each and in all five lobes. There was no lymphadenopathy, parenchymal nodules, tree-in-bud pattern or cavitation.

## Discussion

### Clinical features

Some of the clinical features of the patients in this study differ from those in other South African patients with COVID-19. In a Western Cape study of patients admitted to district hospitals with COVID-19, a female predominance of 58.5% was found.^23^ Similarly, a KwaZulu-Natal study described a female predominance of 52.6%,^20^ while the current study reports a male predominance of 51.3%.

The age of patients admitted was younger than in other similar international studies such as Wong et al. (mean of 46.8 vs. 56 years).^14^ This is likely due to the cohort selection early on in the pandemic, when patients were being admitted based on the inability to self-isolate and not solely on clinical condition requiring medical intervention.

Nine percent of the female patients were pregnant at the time of hospital admission, with at least two patients in their third trimester and one in their first trimester. Three of the patients delivered via normal vaginal delivery during their admission and were subsequently discharged. The other three were discharged while still pregnant.

Seventy-seven percent of admitted patients had at least one comorbidity, significantly higher than in similar studies from other countries such as China (27.0%),^24^ as well as in the study by Mash et al. in the Western Cape (66.0%).^23^ The most recorded comorbidities were diabetes mellitus (35.4% vs. overall diabetes mellitus disease prevalence in the South African population of 11.3%)^25^ and hypertension (35.4% vs. 26.9% in the South African population),^25^ both of which are endemic in South Africa and common comorbidities in patients requiring hospitalisation for COVID-19.^14,26^ The prevalence of HIV in the study sample is higher than that of the general population (16.8% vs. 13.7%),^27^ but lower than in the studies by Moodley et al. (17.7%)^20^ and Parker et al. (21.0%)^28^ although the HIV status of approximately 61.0% of the current study participants was unknown. The significance of HIV in relation to hospitalisation is not known for this cohort given the aforementioned indications for admission; however, a recent meta-analysis performed by Danwang et al.^29^ found patients living with HIV to have an increased risk of COVID-19-related hospitalisation.

### Chest radiograph findings

This study demonstrates radiographic findings that parallel those of published literature in the rest of the world.

Early on in the pandemic, Wong et al.^14^ described the most frequent chest radiograph changes in a study of 64 patients with COVID-19 to be predominantly bilateral, lower zone and peripheral in distribution with consolidation being the most common finding (47.0%), followed by ground glass opacities (33.0%). This was later corroborated by Lomoro et al.^30^ with a cohort of 58 patients, which demonstrated similar findings of bilaterality (78.1%) and lower lobe predominant disease (46.9%), and Toussie et al.,^19^ who demonstrated changes most commonly in the right lower zone (42.0%). These COVID-19 related chest radiograph findings have subsequently been supported by various meta-analyses^11^ and systematic reviews.^9^

In this study, ground glass opacities were documented in 58.4% of chest radiographs, followed by consolidation in 29.2%. As it is now known that consolidation is more commonly seen at a later stage in the disease process, usually occurring in the peak stage of disease between days 10 and 13,^31^ these findings most likely show that this study’s cohort of patients were predominantly admitted in the earlier stages of the disease. In addition, considering that only the first or admission chest radiograph was included in this study, it is possible that the pattern of disease may have shifted towards consolidation predominance on later, serial images given the authors’ subsequent understanding of the temporal changes of COVID-19 pneumonia on imaging.^32^

The most commonly affected lung zone was the right lower zone (49.6% ground glass opacities and 17.7% consolidation), which is concordant with the findings of Toussie et al.^19^ and Zhou et al.^33^ In this cohort, despite only one-fifth of patients being admitted to ICU, the left upper zone was relatively spared with only 7.0% ground glass opacities and 1.8% consolidation, corroborating the unusual local findings of Buckley et al.^21^ A few postulates for these specific disease patterns have been proposed, one of which is that anatomically the bronchus of the right lower lobe of the lung is steeper and straighter than other bronchial branches, with a smaller angle between the right lower lobe and the long axis of the trachea. Therefore, the virus is more likely to enter the branches of the right inferior lobar bronchus and cause infection in this distribution rather than in other lobes.^33,34^

Another theory is that these findings may be explained by the relative hypoperfusion of the left upper lobe when compared to the right, as well as differences in lymphatic drainage.^21^ On a pathogenetic level, SARS-CoV-2 spike proteins bind to the angiotensin-converting enzyme 2 receptor which is expressed in alveolar epithelial type II cells, ultimately resulting in apoptosis of these cells. As viral replication accelerates, the epithelial-endothelial barrier integrity is compromised, and pulmonary capillary endothelial cells are damaged, triggering an inflammatory response resulting in the accumulation of inflammatory cells in the alveoli and air spaces, as well as endothelialitis.^35^ Interstitial inflammatory infiltrates and oedema are detected on imaging as ground glass opacities.^35,36^ Therefore, the differential perfusion of the lung lobes may result in greater disease manifestation in those areas which are better perfused.^21^ In addition, differential lymphatic drainage may result in the differential clearing of inflammatory mediators and cellular debris, contributing further to the differential lobar manifestations.^21^ These theories, however, require further histological and pathological corroboration.

**FIGURE 7:**
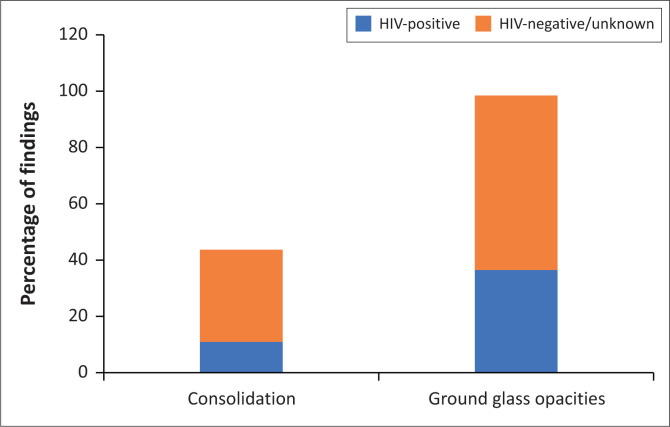
Common COVID-19 related chest radiograph findings in HIV-positive patients compared to HIV-negative patients or patients with an unknown HIV status.

In those patients with HIV, ground glass opacities were detected in 36.0% of the chest radiographs, whereas in the HIV-negative or unknown patients ground glass opacities were detected in 63.0% (*p* = 0.037). Similarly, consolidation was detected in 10.5% of the HIV-positive versus 32.0% of the HIV-negative or unknown patients (*p* = 0.05) (Figure 7). These findings suggest less of a respiratory manifestation of the disease on admission to hospital in the HIV-positive cohort; however, this may be due to clinicians employing a lower threshold for admission of these patients, regardless of clinical condition, given the unknown impact of HIV on COVID-19 outcomes at the time of cohort selection. In addition, this finding is skewed by the large proportion of unknown HIV statuses and thus should be interpreted with caution. Further correlation is required with prospective or serial imaging studies, especially as a study performed recently by the Western Cape Health Department in collaboration with the National Institute for Communicable Diseases found HIV to be associated with increased COVID-19 mortality in a South African context.^37^

In the small subset of pregnant women, the percentage with lung changes on chest radiographs was less than that of their nonpregnant counterparts (50.0% vs. 68.0%); however, given the small numbers, the authors were unable to determine whether this was of significance. The findings of ground glass opacities (33.3%) in a bilateral and lower zone distribution with a similar pattern of consolidation in one study are in keeping with the findings of Wu et al.,^38^ in that the chest radiographic findings of pregnant women with COVID-19 are similar to those of nonpregnant patients.

Only one patient in this study’s cohort had pulmonary TB, with extensive ground glass opacities in all lung zones in both a central and peripheral distribution, as well as central consolidation in all but the right lower zone on chest radiograph. Findings typical for pulmonary TB such as cavitation, lymphadenopathy and/or bronchiectasis were not found. These findings were also inconsistently reported by Moodley et al.^20^ This is likely due to the very low prevalence of TB in both cohorts.

### Computed tomography findings

This study’s cohort of chest CTs was small relative to previous studies; however, the findings were similar to many international studies,^8,14,30,39^ with the most common finding being bilateral ground glass opacities. Unlike Caruso et al. who reported lymphadenopathy in 59% of the CTs in their large cohort, lymphadenopathy was not detected in the current study’s cohort. Consolidation and crazy paving were only detected in one patient each. Architectural distortion, subpleural lines and septal thickening were also identified in one study in the lower lobes predominantly, all of which are signs seen more commonly in the resorptive stage of the disease, indicating local inflammatory absorption with residual fibrosis and associated bronchus distortion.^31,32,40^ Diffuse bronchiectasis with background emphysema was found in one patient with COPD. There were no pleural or pericardial effusions, lung nodules or tree-in-bud pattern to suggest other acute comorbid lung diseases; however, the small number of CTs makes it difficult to determine the significance of these findings.

### Limitations

The major limitations of this study are that it is retrospective, single-centre based and performed early during the COVID-19 pandemic in South Africa. Few CTs were performed, dictated by clinical need, affecting the significance and relevance of the CT findings. Finally, the absence of a control group may also have introduced some bias into the interpretation of the imaging.

## Conclusion

This study has demonstrated that the chest imaging findings of COVID-19 in South Africa are similar to those of the rest of the world; however, some differences were identified among the HIV-positive and HIV-negative or unknown participants. The study corroborated the local finding of relative sparing of the left upper lobe in COVID-19 pneumonia, although further multicentre studies with larger patient cohorts are required to validate this currently unique finding and characterise the evolving spectrum of chest imaging findings of COVID-19 in South Africa.
